# Geographical distance and barriers explain population genetic patterns in an endangered island perennial

**DOI:** 10.1093/aobpla/plw072

**Published:** 2016-10-13

**Authors:** Elisabete F. Dias, M. Moura, H. Schaefer, Luís Silva

**Affiliations:** 1 CIBIO, Centro de Investigação em Biodiversidade e Recursos Genéticos, InBIO Laboratório Associado, Pólo dos Açores, Universidade dos Açores, Rua da Mãe de Deus, Apartado 1422, Ponta Delgada, 9501-801 Açores, Portugal; 2 Plant Biodiversity Research, Technische Universität München, 85354 Freising, Germany

**Keywords:** Azores, conservation, endemic plants, isolation by distance, microsatellites, population genetics, tetraploid

## Abstract

Island plants are frequently used as model systems in evolutionary biology to understand factors that might explain genetic diversity and population differentiation levels. Theory suggests that island plants should have lower levels of genetic diversity than their continental relatives, but this hypothesis has been rejected in several recent studies. In the Azores, the population level genetic diversity is generally low. However, like in most island systems, there are high levels of genetic differentiation between different islands. The Azores lettuce, *Lactuca watsoniana*, is an endangered Asteraceae with small population sizes. Therefore, we expect to find a lower level of genetic diversity than in the other more common endemic Asteraceae. The intra- and interpopulation genetic structure and diversity of *L. watsoniana* was assessed using eight newly developed microsatellite markers. We included 135 individuals, from all 13 known populations in the study. Because our microsatellite results suggested that the species is tetraploid, we analysed the microsatellite data (i) in codominant format using PolySat (Principal Coordinate Analysis, PCoA) and SPAgedi (genetic diversity indexes) and (ii) in dominant format using Arlequin (AMOVA) and STRUCTURE (Bayesian genetic cluster analysis). A total of 129 alleles were found for all *L. watsoniana* populations. In contrast to our expectations, we found a high level of intrapopulation genetic diversity (total heterozigosity = 0.85; total multilocus average proportion of private alleles per population = 26.5 %, *F*_is_ = −0.19). Our results show the existence of five well-defined genetic groups, one for each of the three islands São Miguel, Terceira and Faial, plus two groups for the East and West side of Pico Island (*F*_st_ = 0.45). The study revealed the existence of high levels of genetic diversity, which should be interpreted taking into consideration the ploidy level of this rare taxon.

## Abstract (precis)

Oceanic islands are of particular importance for the conservation of global diversity. Rare island plants with small population sizes and unique genetic patterns resulting from prolonged evolutionary isolation are usually extremely fragile. Therefore, research dedicated to conservation of these plants should consider threat factors, population structure and genetic diversity to enable the development of scientifically supported management programs. For Lactuca watsoniana, Azorean lettuce, we performed a comprehensive population genetic study, unravelling genetic diversity patterns intrinsically related to geographical distances and barriers. Our study showed that genetic diversity levels in island plants can be relatively high even for rare plants.

## Introduction

Oceanic islands have been important study systems for ecologists, evolutionary and conservation biologists and are widely recognized as natural laboratories, for studying evolution due to their discrete geographical nature and diversity of species and habitats (Emerson 2002). Oceanic islands generally have lower overall species numbers per unit area (Whittaker and Fernández-Palacios 2007) but show higher percentages of endemism than mainland areas (Kier *et al*. 2009).

Geological, geographical and ecological conditions, such as island age and area, geographical isolation by oceanic barriers, climatic stability, environmental heterogeneity, high habitat diversity and the absence of competitors, have been pointed out as key determinants of the speciation patterns observed within volcanic archipelagos (Gillespie and Baldwin 2009; García-Verdugo *et al*. 2014; Mairal *et al*. 2015).

Biodiversity of remote islands has arisen through evolution and adaptation of the few initial colonists (Gillespie 2007), with high levels of endemism displaying unique genetic patterns in comparison with continental relatives (Crawford *et al*. 1987; Stuessy and Ono 1998; Emerson 2002; Whittaker and Fernandez-Palacios 2007, Bramwell and Caujapé-Castells 2011; Silva *et al*. 2015; Takayama *et al*. 2015). Differentiation of island populations is further enhanced by intrinsic traits, such as reduced dispersal capabilities, evolutionary innovations, size and reproductive changes (Price and Wagner 2004; Kaiser-Bunbury *et al*. 2010; Hutsemékers *et al*. 2011) which, in addition to ecological opportunity and chance, play a key role in successful island colonization, and often result in adaptive radiation (Gillespie 2007).

Several classical studies have been directed to estimate the genetic diversity of island endemic plants, including the Hawaiian silversword alliance (Baldwin 2009), the Juan Fernandez Islands groups (Crawford *et al*. 2001) and several Canary Island taxa (Francisco-Ortega *et al*. 2000; Caujapé-Castells *et al*. 2010). Recent studies from Macaronesia (Moura *et al*. 2013; Garcia-Verdugo *et al*. 2015) rejected the hypothesis that island populations always have a low genetic diversity due to bottleneck effects, small population size and adaptation to specific ecological conditions (Bouzat 2010; López-Pujol *et al*. 2013; Meloni *et al*. 2015). Similar results had already been obtained in other parts of the world (e.g., *Abies nebrodensis*—Conte *et al*. 2004; *Nothofagus alessandrii*—Torres-Díaz *et al*. 2007; *Cedrus brevifolia*—Eliades *et al*. 2011). These high levels of genetic diversity may have been the result of genetic drift (Dias *et al*. 2014) or might have resulted from changes in ploidy levels (Crawford *et al*. 2009, 2015).

In the Azores, the endemic plant species studied so far showed a considerable range of population genetic patterns: (i) high genetic diversity but low population differentiation—*Picconia azorica* (Martins *et al*. 2013); (ii) relatively low genetic diversity and low population differentiation—*Prunus azorica* (Moreira *et al*. 2013); and (iii) high genetic diversity (with the exception of very small populations) and high level of differentiation—*Juniperus brevifolia* (Silva *et al*. 2011). Some of this supports the hypothesis of a possible Linnean shortfall in the Azorean flora (Schaefer *et al*. 2011; Moura *et al*. 2015*a*), with previously overlooked diversity in several taxa (*Leontodon*, Moura *et al*. 2015b; *Platanthera*, Bateman *et al*. 2013; *Viburnum*, Moura *et al*. 2013).

Among the endemic Asteraceae, the genera *Leontodon* and *Tolpis* have already been studied and showed high population genetic diversity and a complex genetic structure, with clear geographical-linked patterns (Dias *et al*. 2014; Moura *et al*. 2015*b*; Silva *et al*. 2016).

The Azores lettuce (alfacinha), *L. watsoniana*, is a perennial herb, endemic to the Azores, today restricted to four of the nine islands of the archipelago (Faial, Pico, São Miguel and Terceira). It is probably extinct in São Jorge Island, from where two specimens exist in the Lisbon University herbarium (LISU), collected between the valleys of ‘Ribeira do Salto’ and ‘Ribeira de S. João’. *Lactuca watsoniana* is today restricted to the steep slopes of craters, ravines, and temperate juniper rain forest, between 600 and 800 m above sea level (Schaefer 2005; Silva *et al*. 2009; ED, pers. obs.).

Estimates for its total population size range from 500 to 2000 individuals (Schaefer 2005; Silva *et al*. 2009, 2011), but recent field observations indicate lower numbers (probably fewer than 500 individuals). Like many other endemic plants from oceanic islands (Francisco-Ortega *et al*. 2000; Caujapé-Castells *et al*. 2010) it is considered a priority species for conservation and was listed as endangered [EN B2ab(i,ii,iii); C2a(i)] on the IUCN Red List 2013. Furthermore, it was included in Habitats Directive as a Priority species (Annex B-II) and also in Bern Convention (Appendix 1, Annex 1).

The species is threatened by habitat loss and degradation resulting from changes in land use, namely expansion of pastureland, invasion by exotic species, introduced herbivores and disturbance of sensitive areas by tourists and locals (Silva *et al*. 2009). *Lactuca watsoniana* propagules are dispersed by wind and water (Schaefer 2003; Silva *et al*. 2009) but it is unknown, how efficient these strategies are under the specific conditions in the Azores archipelago with predominantly westerly winds and individual islands separated by distances of 6 km (Faial to Pico) to 600 km (Santa Maria to Corvo) of open ocean.

As consequence, the remaining often small and geographically isolated populations on the different islands are likely to have low genetic diversity and could suffer from inbreeding depression (Lowe *et al*. 2005).

Besides the scientific interest related to the study of evolutionary processes in islands and particularly in the Azores, where many scientific gaps still exist (Carine and Schaefer 2010; Schaefer *et al*. 2011; Moura *et al*. 2015b, c), practical issues are also involved when it comes to the conservation of endangered endemic plants. Recently, Silva *et al*. (2015) proposed that population genetic studies should be included in more holistic approaches to research devoted to rare island plants, since different views exist about crucial aspects such as propagule provenance (Edmands 2007; Silva *et al*. 2011; Weeks *et al*. 2011; Dias *et al*. 2014; Silva *et al*. 2016).

In the context of a holistic view, and considering that a previous germination study was already conducted for *L. watsoniana*, we additionally performed a comprehensive population genetic study of *L. watsoniana* populations using newly developed microsatellite (Simple Sequence Repeats, SSR) markers. Based on the relative depauperation of the extant populations, we expected to find relatively low levels of genetic diversity.

Our objectives were (1) to determine the population genetic structure of *L. watsoniana*; (2) to estimate intra and inter-population genetic variation patterns; (3) to identify populations with low genetic variability and inbreeding; and (4) to verify the occurrence of other putative operational taxonomic units.

Beyond the conservation of *L. watsoniana*, we also aim to use the results of the present study to better understand the evolution of island plants in the Azores as well as in other relatively remote and geologically young island systems.

## Methods

### Study site

The Azores Archipelago includes nine volcanic islands, located in the NE of the Atlantic Ocean, between 36° and 43° N and 25° and 31° W, extending for more than 600 km and lying in a northwest–southeast direction. The closest mainland is the Iberian Peninsula, approximately 1363 km East, Newfoundland is 2272 km Northwest, Bermuda 3455 km southwest and Madeira 340 km southeast. There are nine major Azorean islands in three main groups: Flores and Corvo, to the west; Graciosa, Terceira, São Jorge, Pico and Faial in the centre; and São Miguel, Santa Maria and the Formigas Reef to the east. The archipelago surface is c. 2334 km^2^. However, the islands reveal very uneven dimensions: the larger, São Miguel (745.8 km^2^), Pico (448.4 km^2^) and Terceira (403.4 km^2^), represent 70 % of the total surface; São Jorge (245.9 km^2^), Faial (173.8 km^2^) and Flores (141.6 km^2^) have an intermediate size; Santa Maria (97.1 km^2^), Graciosa (61.2 km^2^) and Corvo (17.2 km^2^) are the smallest of the archipelago. Santa Maria is the oldest island of the archipelago (6.3 M years) and Pico being the youngest island of the Azorean Archipelago (0.27 M years; Ávila *et al*. 2016). The Azorean climate is temperate oceanic with a mean annual temperature of 17 °C at sea level, low thermal amplitude, high mean relative humidity, persistent wind and rainfall ranging from 800 to 3000 mm/m^2^, increasing with altitude (Azevedo 1996).

The vascular plant flora is currently thought to comprise c. 1110 taxa, including 73 endemic taxa (Silva *et al*. 2010). However, these numbers likely underestimate the true diversity, since recent molecular studies have repeatedly revealed new endemic taxa (Schaefer and Schönfelder 2009; Bateman *et al*. 2013; Moura *et al*. 2015b, c; Schaefer 2015). Many species introductions and land use changes led to the replacement of natural plant communities, with more than 60 % of the surface today covered by pasture land (Schaefer 2003; Lourenço *et al*. 2011; Costa *et al*. 2012; Marcelino *et al*. 2013).

### Plant material and sampling

A total of 13 different populations and 135 individuals of *L. watsoniana* were sampled, along with a subsample of five individuals of *Lactuca palmensis*, one or two individuals from selected North American species and one individual of *L. sativa* (Table 1). All the places from which *L. watsoniana* populations have been mentioned in historic or recent records, or which were known to local botanists or to the Environmental Services were visited, but only 13 could be confirmed. We searched without success for the only population known from São Jorge and for one population in São Miguel (Lagoa do Fogo, see Table 1).

**Table 1:** Geographical distribution *Lactuca watsoniana* populations sampled in the Azores archipelago, and different herbarium specimens included in this study (*lactuca palmensis* and several Lactuca species from North America). A total of 150 individuals were sampled; (N), total number of individuals per island/location, (n) number of individuals per population. * Population excluded from the analyses (less than 6 individuals).

Species	Location (*N*)	Populations	Codes	*n*
*L. watsoniana*	Faial (11)	Caldeira	FACA	11
	Pico (65)	Prainha	PIAP	13
		Caveiro	PICA	17
		Gruta dos Montanheiros	PIGM	13
		Subida Montanha	PISM	21
		Caldeirão Ribeirinha*	PIRB	1
	Terceira (33)	Grota das Alfacinhas*	TEGA	2
		Ribeira dos Gatos*	TENR	2
		Rocha do Chambre	TERC	13
		Serra do Galhardo	TESG	16
	São Miguel (26)	Caminho Criação*	SMCC	1
		Lagoa do Canário	SMLC	19
		Miradouro Canário	SMMC	6
*L. biennis*	N.A (1)	Cultivated BGBM*	LB	1
*L. sativa*	São Miguel (1)	Mercado Graça*	LSMG	1
*L. palmensis*	Canary Islands (5)	Las palma*	LPLP	5
*L. canadensis*	Dominican Republic (1)	Provincia Santiago*	LC	1
	Minnesota(1)	Hubbard County*		1
*L. floridana*	Florida (1)	Lake Okalumpka*	LFL	1
*L. graminifolia*	South Carolina (1)	Brunwick County*	LG	1
*L. tenerrima*	Malacitona (1)	BM herbarium*	LT	1
*L. hirsuta*	Lousiana (1)	New Orleans*	LH	1
*L. ludoviciana*	Nebraska (1)	Hitchcock County*	LL	1
	South Dakota (1)	Deuel County*		1

**Figure 1:**
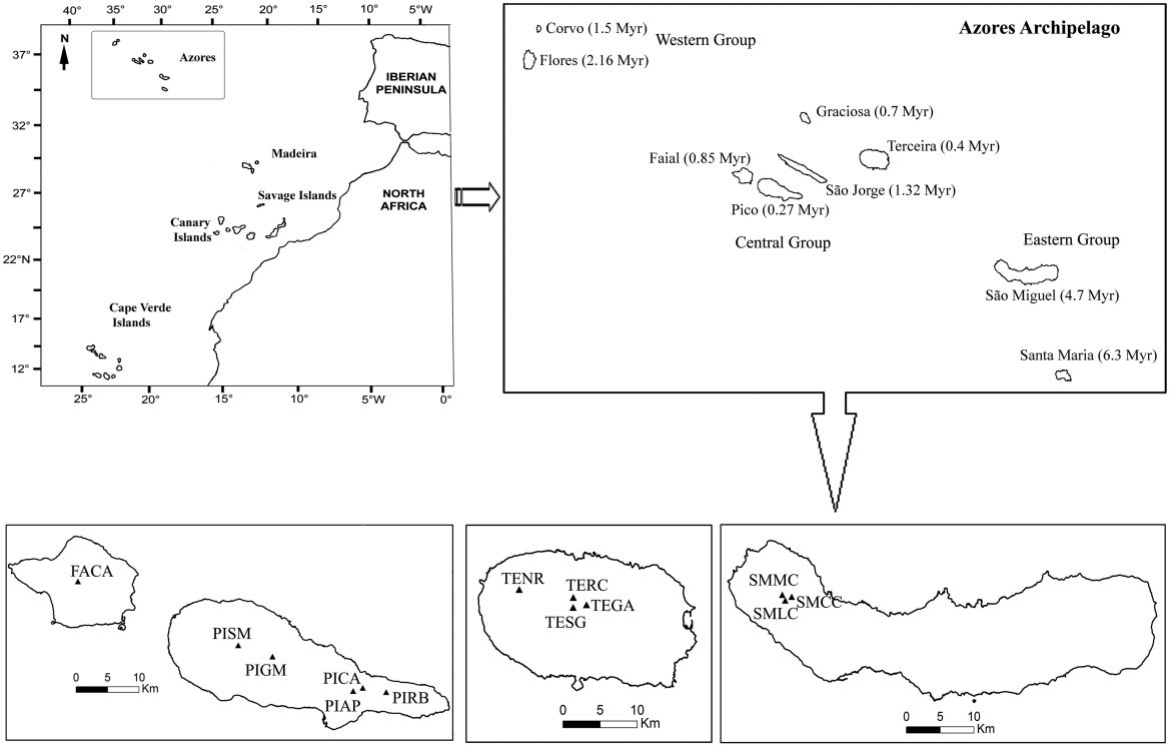
A map showing the Azores archipelago within Macaronesia. Within the rectangle on the right details of the Azores islands are shown, with updated islands ages (Sibrant et al. 2015; Ávila et al. 2016). At the bottom, studied populations of *Lactuca watsoniana* are shown. The symbols and abbreviations indicate sampled sites within each island (Table 1).

In 2012 and 2013, trips to Terceira and Pico islands were carried out in order to complement the samples of *L. watsoniana* already available at the DNA bank collection of the AZB herbarium (Biology Department, Azores University). Samples from every single individual were taken in populations with up to 30 plants and in larger populations, 20 samples were collected in populations. *Lactuca palmensis* from La Palma Island (Canary Islands) and North American species—*L. biennis, L. canadensis, L. floridana, L. graminifolia*, *L. hirsuta, L. ludoviciana* and *L. tenerrima*—were obtained from herbarium specimens of the United States National Herbarium, Smithsonian Institute; *L. tenerrima* samples were also obtained from specimens at the BM herbarium of The Natural History Museum, London; and a *L. sativa* sample was obtained in the local market. Depending on leaf size, we collected one or two leaves per individual and immediately stored them in a plastic bag with silica gel. After drying, the leaves were vacuum sealed in plastic bags and stored in folders. The locations of all populations were geo-referenced and mapped using ArcMap® 10.2.2 (Fig. 1).

### DNA extraction and microsatellites development

DNA was extracted from dry leaves using a modified Doyle and Dickson CTAB protocol (Doyle and Doyle 1987). Due to the difficulties encountered in obtaining high quality DNA, modifications proposed by Borges *et al*. (2009). DNA quality and quantity were measured using a Nanodrop 2000 (Thermo Fisher Scientific) spectrophotometer. Samples were conserved at −20 °C until use.

Total DNA from fresh leaves of two individuals of *L. watsoniana* was sent to the Savannah River Ecology Laboratory (University of Georgia) where an Illumina paired-end (IPE) shotgun library was prepared for microsatellite sequencing (Castoe *et al*. 2012). The resulting sequences were analysed to identify microsatellite repeats for which primers were designed with *PAL_FINDER_v0.02.03*.

Out of the 48 sequences of primer pairs provided by the Savannah River Ecology Lab we selected 24 primer pairs with expected PCR products ranging between 100 and 400 bp to allow multiloading of PCR products (Oetting *et al*. 1995). All the primer pairs (with the tag sequence included) were selected on criteria of non-complementarities within and between primers, low secondary structures and 3′-end instability (Rychlik 1995).

One primer from each pair was extended on the 5′-end with an engineered sequence (M13R tag 5′-GGAAACAGCTATGACCAT-3′) to enable the use of a third primer identical to the M13R, which allows for an inexpensive fluorescent labelling of the PCR product obtained (Schuelke 2000). A GTTT ‘pigtail’ was added to the 5′-end of the untagged primer to facilitate accurate genotyping (Brownstein *et al*. 1996).

### Microsatellite selection

In the first phase of the test, all 24 primer pairs were tested on four samples of *L. watsoniana* using a final volume of 25 μl, including 25 ng of DNA, 1× NH_4_ Buffer, 1.5 mM MgCl_2_, 0.4 μM of FAM-M13R, 0.08 μM of tagged primer, 0.36 μM of unlabelled tag primer, 200 μM of dNTPs, 1U of Polymerase (Immolase, Bioline) and using a Biometra TGradient thermocycler. Touchdown thermal cycling programmes (Don *et al*. 1991) encompassing a 10 °C span of annealing temperatures, ranging between 64 and 54 °C, were used for all loci. The PCR program consisted of the following steps: 95 °C for 7 min (hot start); 95 °C for 3 min; 20 cycles at 95 °C for 30 s, the highest annealing temperature of 65 °C (decreased by 0.5 °C per cycle) for 30 s, and 72 °C for 30 s; 20 cycles of 95 °C for 30 s, 55 °C for 30 s and 70 °C for 30 s; and finally, 72 °C for 10 min for the final extension of the PCR products. Five microliters of PCR products were run on a 3.5 % agarose gel, stained with SafeView™ Nucleic Acid Stain (abm) and visualized under UV light to check for amplification, polymorphism and scorability of the bands. Eleven primer pairs exhibited scorable amplified products of the expected length range and with at least two alleles. In the second phase, the variability of the 11 polymorphic loci was assessed in 13 samples, one from each population. After analysis of the quality of the PCR products obtained with the universal primer M13R, eight primer pairs with acceptable to high scorability were selected to run the complete study (Table 2).

**Table 2:** Description of the eight SSR polymorphic loci selected polymorphic SSR loci used for genetic discrimination of the Azorean *Lactuca watsoniana* populations. ^a^Indicates M13R tag (5′-GGAAACAGCTATGACCA-3′). ^b^Indicates ‘pigtail’ tag (5′-GTTT-3′).

Name	Primer	Repeat Motif	Sequences	Total alelles (#)	Size Range (bp)	Dye
Lw-01.06093	Forward	(TTC)^60^	^a^GGAAACAGCTATGACCATAAGAAGAAGAGTTGGTGCTGTCG	18	150–200	FAM
Lw-01.10034	Reverse		^b^TGTTCTCCGCGTTGTTTGG			
Lw-01.01134	Forward	(TTC)^48^	^a^GGAAACAGCTATGACCATCAGGAAGGATAATTGGTCTCTTAGG	15	230–300	FAM
Lw-01.05248	Reverse		^b^AGCACCAGGTGAGATCAAGG			
Lw-01.01742	Forward	(TTC)^57^	^a^GGAAACAGCTATGACCATCCCTGTGTTACAACCACCC	31	230–320	VIC
Lw-01.01412	Reverse		^b^CCATGTTAATGGCATTGTATCC			
Lw-01.09032	Forward	(ATAC)^36^	^a^GGAAACAGCTATGACCATCATAACCTGCCTACAGAAGAGGG	15	230–310	NED
Lw-01.02225	Reverse		^b^GATTGTTATATCATGAATTTGACTGAAAGG			
Lw-01.02384	Forward	(ATCT)^48^	^a^GGAAACAGCTATGACCATTCTACGTGAGTTCACAATTTCACC	15	160–210	PET
Lw-01.05506	Reverse		^b^TGATGCTGTAGAAGATGCTGC			
Lw-01.03892	Forward	(ATT)^39^	^a^GGAAACAGCTATGACCATTGTATTATTTCTTTATGGTGATTCCC	18	240–300	PET
Lw-01.09716	Reverse		^b^TTAAGCTTAGGACGAGAAAGTCG			
Lw-01.03674	Forward	(ATT)^42^	^a^GGAAACAGCTATGACCATGAGAATCGGTCTACTAATGCAGC	10	140–180	PET
Lw-01.04032	Reverse		^b^CATAACCAACACCAATGCCC			
Lw-01.08037	Forward	(ATAC)^32^	^a^GGAAACAGCTATGACCATCCCATCAACAAAGTGATTAGGC	17	300–370	PET
Lw-01.08435	Reverse		^b^CATGAGCATGAGTGGTTCTCC			

### Full-scale genotyping

After optimization, all samples were amplified following Dias *et al*. (2014). The M13R was labelled either with PET, FAM, NED or VIC. The Taq polymerase used with all the markers was Immolase (Bioline). Amplification products were diluted, multiloaded, run on an ABI-3130xl Genetic Analyser and sized with LIZ500 size standard. The genotypes obtained were scored using the software Geneious R8 (http://www.geneious.com; Kearse *et al*. 2012). The population matrix is available at DEMIURGE (http://www.demiurge-project.org/) with digest code D-CPMIC-101.

### Data analyses

SSR preliminary analyses showed a pattern compatible with tetraploidy since all individuals displayed four alleles per locus. Cytological analysis of seedling root tip material later confirmed the tetraploid nature of *L. watsoniana* (manuscript in prep). This information was used to define the statistical analysis methods, since polyploid organisms raise analytical limitations, namely, problems associated with inferring allele frequencies and assumptions regarding inheritance (Obbard *et al*. 2006; Dufresne *et al*. 2014).

#### Genetic diversity

Considering the tetraploid nature of *L. watsoniana*, we used specific software to generate the genetic dataset. Therefore, genetic diversity for each population was calculated using a codified co-dominant matrix with SPAGeDi (Hardy and Vekemans 2002), as a multilocus average for the eight loci, including the number of alleles (NA), the allelic richness (AR), Nei’s gene diversity (1978) (He), global inbreeding coefficient (*F*_Is_), computed for all loci and individual inbreeding coefficient (*F*_i_), computed as kinship coefficient between homologous gene copies within individuals. The total genetic diversity (H_T_) was determined using the unbiased estimate method of Nei (1987). The percentage of private alleles was calculated using Microsoft Excel^®^ based on SPAGeDi allelic frequency data.

#### Population structure

Taking into consideration the ploidy level, we first analysed the population structure with a Principal Coordinate Analysis (PCoA), based on the matrix of Bruvo distances between individuals, using the R package PolySat (Clark and Jasieniuk 2011). Then we used a Bayesian approach estimate the number of genetic clusters excluding populations with fewer than six plants (PIRB, SMCC, TEGA and TENR) which resulted in a final number of 129 individuals in the analyses. The data were exported from PolySat to a dominant matrix (presence/absence of alleles), which was run with the program STRUCTURE version 2.3.4 (Pritchard *et al*. 2000; Kumar *et al*. 2009). We followed the admixture model along with the assumption of correlated allele frequencies between groups (Falush *et al*. 2003), with 500 000 MCMC repetitions, exclusion of a burn-in period of 50 000 and 10 replicates for each *K* value ranging from 1 to 10. Estimation of best *K* value was conducted with STRUCTURE Harvester (Earl and von Holdt 2012) following the approach of Evanno *et al*. (2005). The optimal *K* repetitions were permuted in Clumpp version 1.1.2 (Jakobsson and Rosenberg 2007) using the Greedy algorithm and results were graphically represented using Distruct version 1.1 (Rosenberg 2004). For Analysis of Molecular Variance (AMOVA), we used the dominant matrix and Arlequin 3.5 (Excoffier *et al*. 1992; Excoffier and Lischer 2010) to (1) calculate the partition of genetic variability within populations, among populations within islands, and among islands and (2) estimate gene flow among populations and the global Wright index, *F*_st,_ under the null hypothesis of Hardy–Weinberg (HW) equilibrium.

#### Geographical barriers and isolation by distance

To determine the order of barriers to gene flow possibly occurring within the archipelago, the Nei’s genetic distance matrix corresponding to the Azorean populations was analysed with the Monmonier algorithm, using BARRIER version 2.2 (Manni *et al*. 2004) and allowing a maximum number of nine barriers (the total number of populations analysed after excluding four populations with fewer than six individuals). Nei’s genetic distance (Nei 1972, 1978) was calculated using a dominant matrix with GenAlEx 6.5 (Peakall and Smouse 2012).

We followed the approach of Meirmans (2012) to discern between a Hierarchical Island model and a Stepping Stone model using standard, stratified and partial Mantel tests using the package VEGAN (Oksanen *et al*. 2009) in R (http://www.r-project.org/). We also modelled Nei’s genetic distance as a function of geographical distance and of the presence of geographical barriers, using beta regression with the BETAREG package in R (Cribari-Neto and Zeileis 2010). We compared the model based on geographical distance alone with models including an increasing number of geographical barriers. We defined the geographical barriers in two ways: (i) considering the existence of barriers between the different clusters identified with STRUCTURE; and (ii) considering the existence of barriers between populations as determined with BARRIER. Model fit was compared using AIC and pseudo-*R*^2^.

## Results

### Genetic diversity

The eight developed primers showed acceptable to high scorability results, with exception for the *L. sativa* sample (all loci) and *L. biennis* (loci Lw-01.08037 and Lw-01.08435). A total of 129 alleles were found for *L. watsoniana*, with a total genetic diversity (H_T_) of 0.85, an overall excess of heterozygotes (Multilocus *F*_is_ = −0.1912, *P* < 0.001; Average Multilocus *F*_i_ values = −0.074, *P* < 0.001), and with a total multilocus average proportion of private alleles of 26.5 %. The total number of alleles per locus varied from 10 to 31 (Table 2).

The ‘Lagoa do Canário’ population of São Miguel Island displayed the highest percentage of private alleles (7.0 %) and the highest multilocus number of alleles (9), while the ‘Miradouro do Canário’ population in São Miguel Island and ‘Subida da Montanha’ in Pico Island did not show any private alleles (Table 3). The mean allelic richness ranged from 4.58 alleles in the ‘Subida da Montanha’ population up to 6.18 alleles in the ‘Lagoa do Canário’ population. The populations showed similar values for gene diversity (0.7 and 0.8, Table 3).

**Table 3:** Genetic diversity parameters based on eight SSR loci within nine populations of *Lactuca watsoniana*, with multilocus average. *Abbreviations*: number of samples (*N*), number of alleles (NA), allelic richness (AR), gene diversity (He) and inbreeding coefficient (*F*_i_), ignificance levels (Pv: *P < 0.01; **P < 0.05), and average percentage of private alleles (Pa).

Island	Populations	*N*	NA	AR	He	*F*_i_	Pv	Pa
Faial	Caldeira	11	7	5.66	0.79	−0.27	*	1.69
Pico	Prainha	13	8	6.07	0.80	−0.14	*	4.55
	Caveiro	17	7	5.46	0.77	−0.17	*	1.72
	Gruta dos Montanheiros	13	6	4.97	0.74	−0.18	*	5.88
	Subida Montanha	21	6	4.58	0.71	−0.15	*	0.00
Terceira	Rocha do Chambre	13	6	4.95	0.74	−0.35	*	1.96
	Serra do Galhardo	16	7	4.86	0.76	−0.26	*	3.64
São Miguel	Lagoa do Canário	19	9	6.18	0.82	−0.16	*	7.04
	Miradouro Canário	6	6	5.52	0.80	−0.10	**	0.00
Total		129	15.88	7.38	0.85	−0.07	*	26.49

### Population genetic structure

**Figure 2:**
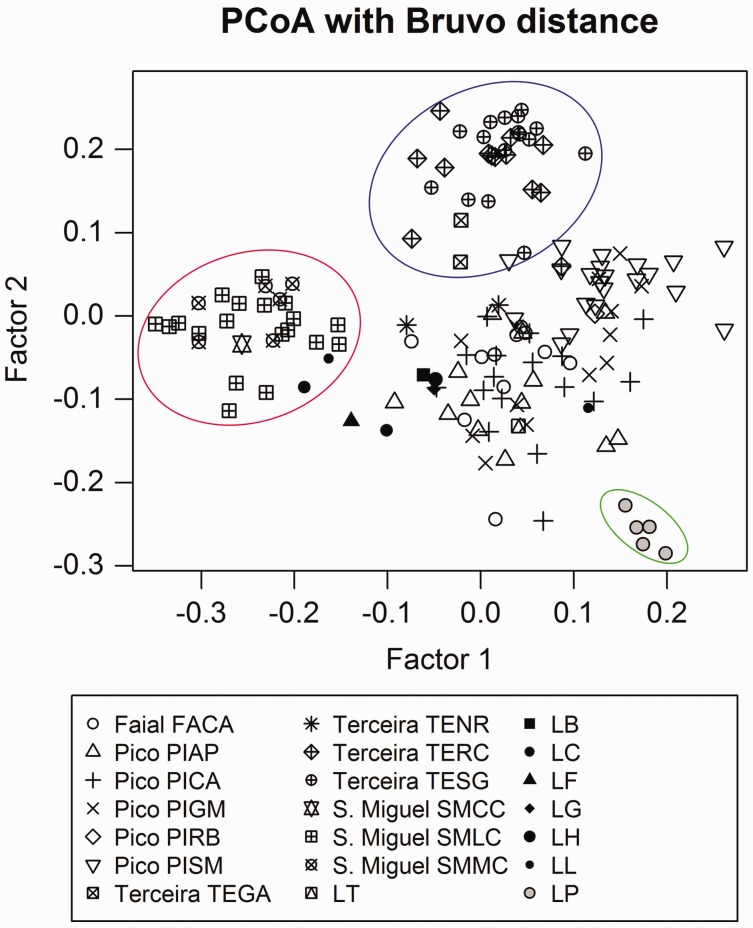
Principal Coordinate Analysis (Bruvo distance) based on the data set of nine microsatellite loci for all 148 *Lactuca* individuals included in this study. Inertia percentage of axes 1 (13.72 %) and 2 (10.41 %). Each individual is represented by a symbol according to its geographical distribution (sampling site labels as in Table 1). Red ellipse limits the individuals from Sao Miguel, individuals from Terceira are limited with a blue ellipse and *L. palmensis* in the lower right part of the chart are limited by a green ellipse.

**Figure 3:**
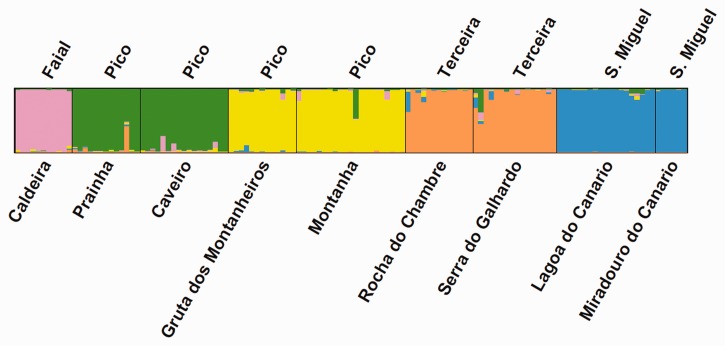
Results of genetic admixture analysis performed with STRUCTURE (*K* = 5, genetic clusters indicated in different colours) based on the SSR loci scored for *L. watsoniana*. Each vertical bar corresponds to an individual, populations are indicated below the graphic and islands, above.

The initial analysis of 148 samples (all except *L. sativa* e *L. biennis* samples) from 13 populations, based on eight microsatellite loci, revealed the existence of a close relationship between the North American species and *L. watsoniana* populations and a clear separation from *L. palmensis*, as shown by PCoA (Fig. 2). *Lactuca watsoniana* individuals were also separated according to their geographical location, but with one cluster combining the populations of the neighbouring islands Faial and Pico (Fig. 2). A Bayesian analyses, showed a peak of Δ*K* for *K* =  5. Taking into consideration, the variation across runs, and the partitioning of individuals, we assumed *K * = * *5 as the most likely value. The results obtained with STRUCTURE (Fig. 3) show a non-admixture pattern with the definition of five genetic clusters corresponding to each of the islands of origin (Faial, Terceira and São Miguel), and to the separation of the four Pico Island populations into two genetic clusters (PIAP and PICA, more to the west versus PIGM and PISM, more to the east). This result is in accordance with those obtained with PolySat (Fig. 2) and GenAlEx 6.5 (data not shown). The AMOVA analyses applied to the groups defined above showed that the largest proportion of genetic variation was found within populations (55.0 %) but with relatively high levels of genetic variation both among clusters (27.3 %) and (17.68 %) among populations within clusters (Table 4). Global *F*_st_ value calculated (0.45) denoted a considerable overall differentiation among populations, with *F*_st_ values ranging from 0.13 between São Miguel populations (SMLC and SMMC) up to 0.99 between PICA and TERC populations (Table 5). Gene flow values calculated between populations indicated a relatively high number of migrants between some of the geographically close populations within São Miguel and Pico islands (Table 5), but were much lower for the remaining cases.

**Table 4:** Analysis of molecular variance (AMOVA) conducted on SSR profile of nine populations of *Lactuca watsoniana* from São Miguel, Terceira, Faial and Pico. The clusters were identified with STRUCTURE, *P* < 0.001 for all components.

Populations	FACA	PICA	PIAP	PIGM	PISM	TESG	TERC	SMMC	SMLC
FACA		0.54	0.53	0.65	0.85	1.00	1.54	1.23	0.96
PICA	0.46		0.22	0.34	0.37	0.95	0.99	0.84	0.64
PIAP	0.47	1.11		0.38	0.44	0.78	0.72	0.63	0.54
PIGM	0.39	0.74	0.66		0.35	1.07	1.13	1.09	0.81
PISM	0.29	0.67	0.57	0.70		1.25	1.14	1.16	0.81
TESG	0.25	0.26	0.32	0.23	0.20		0.70	1.43	1.08
TERC	0.16	0.25	0.34	0.22	0.22	0.36		1.28	0.93
SMMC	0.20	0.30	0.40	0.23	0.22	0.17	0.20		0.13
SMLC	0.26	0.39	0.46	0.31	0.31	0.23	0.27	1.94

**Table 5:** Genetic differentiation between pairs of Azorean *Lactuca watsoniana* populations. *F*_st_ values (above diagonal) and gene flow values calculated using the Slatkin formula (below diagonal).

Source of variation	*df*	Sum of squares	Variance component	Percentage of variation (%)
Among clusters	4	562.36	3.82	27.30
Among populations within clusters	4	165.12	2.47	17.68
Within populations	120	924.34	7.70	55.02
Total	128	1651.81	14.00	

### Geographical barriers and isolation by distance

**Figure 4:**
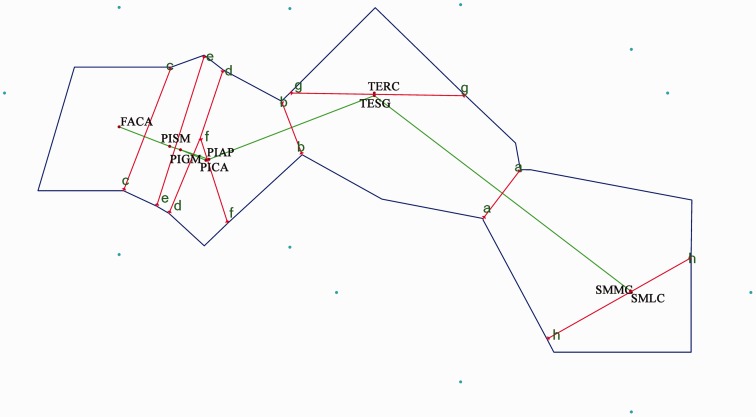
Barriers to gene flow possibly present between nine populations of *L. watsoniana* from São Miguel, Terceira, Faial and Pico (Azores archipelago), calculated by the Monmonier’s algorithm (1973) with the program BARRIER. Barriers are labelled from a to h, alphabetically ordered by relevance.

The first-order barrier to gene flow was computed between São Miguel Island and the islands of the Central Group, while the last barrier was located between the two populations of São Miguel (Fig. 4).

**Figure 5:**
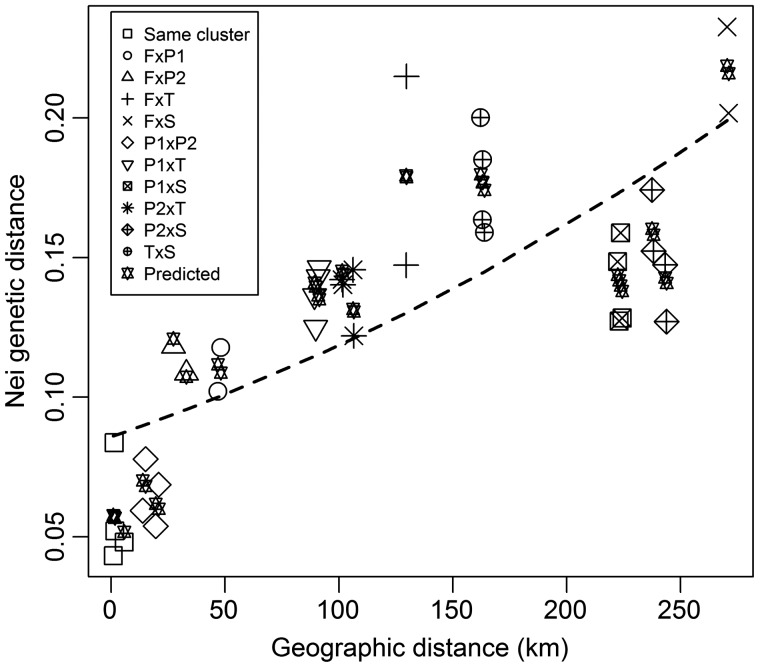
Correlation between pairwise genetic distance (Nei) and geopraphical distance (m) in the studied Azorean Islands The dashed line corresponds to the geographical model only (AICc  = −140.40, pseudo-*R*^2 ^=^ ^0.58). ‘Predicted’ correspond to the genetic distances predicted by the best model, including geographical distance and barriers (AICc  = −168.5, pseudo-*R*^2 ^=^ ^0.92), as defined by the five STRUCTURE genetic clusters. The symbols represent comparisons between populations within the same genetic cluster (Same cluster), or within different genetic clusters (e.g. ‘F × P1’). Abbreviations of STRUCTURE genetic clusters correspond to: F =  Faial (1 population); P1 = Pico (2 populations); P2 = Pico (2 populations); T = Terceira (2 populations); S = São Miguel (2 populations).

Regarding isolation by distance (IBD) models, *L. watsoniana* seems to correspond to an intermediate case between hierarchical island and stepping stone models, with no clear discrimination based on the results obtained from standard, stratified and partial Mantel tests (Table 6). Globally, the Mantel test indicated that there was a significant correlation between genetic distance and geographical distance (*r* =  0.77, *R*^2^ = 0.66, Fig. 5). However, model fit increased from the model including geographical distance alone (AIC = −140.40, pseudo-*R*^2 ^= 0.58) to the models including an increasing number of geographical barriers: (i) considering the existence of barriers between different populations clusters as determined with STRUCTURE (best model including all the barriers, AIC = −168.5, pseudo-*R^2^* = 0.90); (ii) considering the existence of barriers between populations as determined with BARRIER (best model including the first to the third order barriers, AIC = −161.5, pseudo-*R*^2^ = ^ ^0.84). The graphical representation of the best fitting model shows how the inclusion of barriers increases the fit of the geographical model (Fig. 5). Also, on average, Pico populations seem to be genetically closer to all the other populations (Fig. 5).

**Table 6:** Results of standard, stratified and partial Mantel tests distinguishing the effects of isolation by distance (Stepping-stone model—SSM) and hierarchical (hierarchical island model—HIM) population structure following Meirmans (2012, Table 1). ^a^Both models can originate the observed result. ^b^A non-significant result supports HIM. ^c^A significant result with relatively high *r* supports SSM. ^d^A significant result supports HIM, *P* (level of significance of the models).

Matrix A	Matrix B	Adjustment	Mantel’s *r*	*P*	Support to HIM/SSM
Genetic	Geographical	–	0.77	0.0002	No discrimination^a^
Genetic	Geographical	Stratified: permuted with clusters	0.77	0.8125	HIM^b^
Genetic	Geographical	Partial: clusters as covariate	0.69	0.0005	SSM^c^
Genetic	Clusters	Partial: geography as covariate	0.35	0.0364	HIM^d^

## Discussion

### Implications of tetraploidy

The SSR profile obtained in this study supported that *L. watsoniana* is tetraploid. To the best of our knowledge, only two of the nine endemic Azorean Asteraceae are polyploid, namely, *Pericallis malvifolia* (Jeffrey 1992; Jones *et al*. 2014*)* and *L. watsoniana* while seven are diploids: *Bellis azorica*, *Leontodon filii*, *L. hochstetteri*, *L.rigens*, *Solidago azorica*, *Tolpis azorica* and *T. succulenta* (Lack 1981; Fiz *et al*. 2002). In the genus *Lactuca*, Feráková (1977) distinguished three main groups based on chromosome number: the first group with *n* =  8 includes perennial species from Europe and the Himalayas; the second group with *n* =  9 comprises the majority of the taxa from Europe, Mediterranean, Middle East, Africa and India; and the third group includes North American species with *n* =  17, considered to be of amphidiploid origin (Doležalová *et al*. 2002). The ploidy level of *L. watsoniana* was previously unknown (Feráková 1977; Lack 1981). Our results suggested by the SSR pattern observed, point to a closer phylogenetic relationship with the North American taxa.

In the Canary Islands, species-rich lineages like the Asteraceae originated from diploid colonizers (Suda *et al*. 2003), while in other archipelagos (Galapagos Islands, Hawaiian Islands, Robinson Crusoe Islands and St. Helena) the endemic diversity apparently originated from polyploid colonists (Harbaugh 2008; Crawford *et al*. 2009). In the Azores Islands, ploidy levels for most endemic colonizers have not been studied yet, although many taxa are now diploid (Bateman *et al*. 2013; Dias *et al*. 2014). However, tetraploids have been recently found for *Prunus lusitanica* ssp. *azorica* (Moreira *et al*. 2013)*.* It was hypothesized that diploid colonizers might be more common in archipelagos closer to continental source areas while successful polyploid colonizers could be more frequent at more remote islands (Carine *et al*. 2004, Crawford *et al*. 2009). However, at this stage, the number of detected polyploids in the Azorean flora does not support such a claim.

Two possible ancestral colonization events may have occurred in the case of *L. watsoniana*: (a) an allotetraploid colonizer from North America or (b) an ancestral diploid population leading to an autotetraploid form, with later extinction of the ancestral diploid. Considering the absence of other endemic or native *Lactuca* species in the Azores, and the existence of allotetraploid North American *Lactuca* species (Doležalová *et al*. 2002) closely related with *L. watsoniana* (Fig. 2), an early colonization from North America seems a more plausible hypothesis. This is a very rare colonization pathway for vascular plants in the Azores, but was confirmed for another endemic Asteraceae, the Azores goldenrod *Solidago azorica* (Schaefer 2015). Therefore, *L. watsoniana* is not likely to be a neopolyploid, although formation of new cytotypes and their demographic establishment have been recorded for ocean islands (Crawford *et al*. 2009; Stuessy *et al*. 2014). More comprehensive morphological and molecular phylogenetic analyses are currently underway to verify this hypothesis.

### Genetic diversity

The genetic variability found in the *L. watsoniana* populations was comparably high to that found for other Azorean herbaceous endemic taxa (Dias *et al*. 2014; Silva *et al*. 2015), and we found no evidence for inbreeding, despite the population decline that most likely took place in the past five centuries. It might also be a direct expression of polyploidy which causes an increase in gene dosage at the genome. Polyploid taxa often contain higher levels of genetic variation to their diploid relatives (Obbard *et al*. 2006; Garcia-Verdugo *et al*. 2015).

This high diversity of tetraploid colonizers together with self-compatibility would have been an advantage in the establishment of a sexually reproducing population (Crawford and Stuessy 1997, Crawford *et al*. 2009), providing favourable conditions for establishment in one island with subsequent dispersal to other parts of the archipelago. However, studies addressing Azorean endemic plant reproductive strategies are very limited (see Crawford *et al*. 2015 for *Tolpis* spp.). Meanwhile, field and laboratory observations indicate that isolated *L. watsoniana* individuals flower regularly but mostly produce infertile seeds whereas in relatively large populations, such as those found in Pico Island, a very high number of viable seeds are produced, annually. In this sense, according to Ferrer and Good-Avila (2007), the basal members of the tribe Cichorieae are dominated by self-compatibility or partial self-incompatibility exemplified by members of the genera *Cichorium* and *Lactuca*. Further studies should be undertaken to enlighten this matter, namely experiments of forced self-pollination and cross-pollination (Zhang *et al*. 2015).

### Population genetic structure

We found five genetic groups in *L. watsoniana*, supported by AMOVA, PCoA and Bayesian model-based clustering. This genetic structure coincides spatially with the studied islands, with the exception of Pico Island, which is home to two genetic groups. The average *F*_st_ value (0.45) was higher than those found for many other plant species (Bottin *et al*. 2005; Lucio 2011; Chaves 2013). We also observed that genetic variation was mainly found within populations (55.0 %), which is in accordance with the results obtained for *Leontodon filii* and *L. rigens* (Dias *et al*. 2014) and in contrast with Canary Islands endemic species, where most genetic variation was found between populations (*G*_st_ = 77.3 %) (Francisco-Ortega *et al*. 2000). However, a relatively high genetic variation was found among populations within islands (17.7 %), only surpassed in the Azores by the dioecious *Juniperus brevifolia* (Silva *et al*. 2011). An even larger proportion of variation was found among islands (27.3 %), an extreme case in the Azores so far.

Estimated gene flow values were always below 1, contrary to the much higher values obtained for *Leontodon* (Dias *et al*. 2014), but similar to the values obtained for *Viburnum trelease*i (Moura *et al*. 2013), although the latter study was conducted with dominant markers. The highest gene flow value (1.94) between two São Miguel populations (Table 5), and between two Pico populations (1.11), likely reflect their geographic proximity and the absence of relevant geographical barriers. The results of our *F*_i_ analysis did not reveal any evidence for inbreeding. A high proportion of private alleles were found in São Miguel (7.04 %), Pico (5.88 %) and Terceira (3.64 %), indicating that some degree of differentiation occurred between those islands.

### Geographical distance and barriers

*Lactuca watsoniana* show a strong geographic structure. Isolation by distance (IBD) and the existence of effective barriers to gene flow, the Atlantic Ocean and Pico Mountain, were found to be related with genetic distance. Therefore, *L. watsoniana* seems to be an intermediate case between hierarchical island and stepping stone models (Table 6) in what could be seen as an approximation to a two or three dimensions Kimura and Weiss (1964) stepping stone model, complicated by inexistent stones or by insurmountable barriers, i.e. the sea. This may be due to the existence of regional structuring (Morand *et al*. 2002), since oceanic barriers are, in general, more effective than topographic barriers at promoting isolation in insular systems (Gillespie and Clague 2009).

*Lactuca watsoniana* diaspores disperse by anemochory and hydrochory (Schaefer 2003; Silva *et al*. 2009), which are more efficient in small distance than in long distance dispersal. In this case, plants in spatially closer populations will be more similar genetically than those from more distant populations (Meirmans 2012). A limitation in dispersal ability is expected to be a key factor in geographical isolation (Kisel and Barraclough 2010), with a significant impact on relatedness within plant taxa (Papadopulos *et al*. 2011).

### Disturbance from volcanism

After birth and growth of volcanic islands, destructive events such as secondary eruptions, landslides and thick volcanic deposits on the islands surface, might reshape the islands flora due to a massive population extirpation by soil sterilization (Mairal *et al*. 2015; Borges Silva *et al*. 2016), and lead to habitat fragmentation and genetic isolation of populations, which in due course could lead to differentiation and speciation within or among-islands (Gillespie and Roderick 2014; Mairal *et al*. 2015).

Considering our results, differentiation within Pico populations, grouped into two clusters, might be a consequence of the existence of Pico Mountain, acting as a natural geological barrier.

On the other hand, besides geographical distance and the existence of barriers, population genetic structure might be the result of the populations sampled being relicts, resulting from geological events, such as volcanic eruptions, as hypothesized for *Juniperus brevifolia* and *Tolpis azorica* (Silva *et al*. 2011, Borges Silva *et al*. 2016) as an explanation of the population genetic patterns found in Terceira Island. ‘Sete Cidades’ and ‘Lagoa do Fogo’ have been among the most active volcanic systems in São Miguel Island during the past 5000 years (Booth *et al*. 1978; Gaspar *et al*. 2015), where the most recent explosive eruption occurred just before human colonization in the 15th century (Ferreira *et al*. 2015). In Pico, Macedo (1981*a*, *b*) and Machado (1962) documented four major volcanic eruptions in the 16th and 18th centuries, in ‘Prainha’ (1562), ‘Santa Luzia, São João’ (1718) and ‘Silveira’ (1720). These episodes, with deposits of thick pumice, might have exterminated nearby populations of *L. watsoniana*, contributing to the unique observed patterns of genetic variation. Implications of island age on the observed genetic patterns are difficult to determine at this stage, due to the possibility of the later occurrence of volcanic disturbance, as illustrated above, and to a considerable effect of human disturbance (see below). Moreover, during a preliminary analysis, we included island age and differences in island age between populations, in our models to explain genetic distance, but without significant improvement of the modelling results.

### Anthropogenic disturbance

Human colonization has had a strong impact on oceanic islands endemic plants since pristine ecosystems have evolved or persisted in the absence of novel or exotic disturbances (Connor *et al*. 2012). Human population settlement on previously pristine ecosystems, with large scale changes in land use, originated habitat loss and fragmentation both of which contribute to a decline in biological diversity (Wilcox and Murphy 1985; Kuussaari *et al*. 2009; Krauss *et al*. 2010; Triantis *et al*. 2010). In the Azores, widespread and persistent vegetation and soil use changes in the last 500 years, together with the introduction of alien plants (Silva and Smith 2004; Connor *et al*. 2012; Costa *et al*. 2012), and herbivores (Moura *et al*. 2013; Dias *et al*. 2014; Silva *et al*. 2015), led to a decline in native vegetative cover and in the number of endemic plant populations throughout the archipelago. Our field work allowed us to directly observe the results of herbivore consumption on *L. watsoniana* individuals, from almost complete consumption of above ground plant material (goats in São Miguel Island) to the removal of the developing synflorescence (cattle in Terceira Island). Damage caused by human intervention in the native vegetation is the most probable explanation for the possible extinction of *L. watsoniana* in São Jorge Island, where the native vegetation has been replaced by invasive plants in the site of the historical collection. Pico Island holds the highest number of individuals including many young plants, what could be a direct consequence of being one of the less altered islands in the archipelago. One justification would be that Pico represents a sample of a formerly wider genetic pool that was eroded in the remaining islands, which through possible genetic bottlenecks only kept part of that wider pool, similarly to what might have happened to *Tolpis azorica* populations in the central sub-archipelago of the Azores (Borges Silva *et al*. 2016). Whether this actually happened and whether it was related with volcanic or human disturbance remains to be ascertained. Bottleneck analyses are possible for diploid species, but there are no analysis tools presently available to test them in tetraploids (Espinosa and Noor 2002). Therefore, although the impact of human disturbance was observed in most populations, at this stage *L. watsoniana* populations managed to maintain a high degree of genetic diversity and low levels of inbreeding. However, the continuation of this level of disturbance might still lead to more pronounced population decline with unpredictable consequences on future diversity levels.

### Implications in conservation

Our results, of a high genetic diversity, with substantial population differentiation among islands, strongly advocate that the concept of propagule provenance should be taken into consideration when developing augmentation or reintroduction strategies (Silva *et al*. 2011; Weeks *et al*. 2011; Dias *et al*. 2014; Borges Silva *et al*. 2016). For example, the heterogeneity between the western and eastern populations in Pico means that seeds or individuals from the eastern part of this island should not be used in reintroduction programs on the western part of the island or even on the other islands. Since the species shows a considerable degree of genetic diversity, high seed production and high germination rate (Dias *et al*. 2015), some of the conservation actions that should be implemented or established are: (i) running a seed bank and a plant nursery (Attorre *et al*. 2011), where plants from the different islands could be produced following specific protocols for *L. watsoniana* (Dias *et al*. 2015); (ii) reinsertion of the plantlets in the original natural populations, increasing the number of effectives and preserving the genetic diversity. Inter-island and inter-population transfers of seedlings and plantlets should be avoided at all cost, since differentiation levels are high but inbreeding was not found (Moura *et al*. 2013; Dias *et al*. 2014; Borges Silva *et al*. 2016).

The uniqueness of some of the studied populations and the existence of populations with extremely low number of individuals, indicate that the populations of Pico (PIRB, PIAP, PICA, PIGM and PISM), ‘Caminho da Criação’ on São Miguel (SMCC) and Terceira (TEGA and TENR), should be considered priority for conservation.

The awareness of the threats by habitat loss and degradation resulting from changes in land use indicates that extreme care should be taken regarding the implementation of augmentation conservation strategies. Some measures have been implemented to locally control invasive species (Costa *et al*. 2013). However, *L. watsoniana* populations in São Miguel island are severely threatened by herbivores (feral goats and rabbits), which in Terceira have already alarmingly obliterated most of the Serra do Galhardo population. These animals should be controlled or eradicated from these protected areas.

## Conclusion

Our research underscores the importance of considering a broad array of intrinsic traits and external factors when studying the evolution and management of plants in oceanic islands.

Regarding intrinsic traits, our results support previous claims suggesting that high levels of genetic variation are often associated with polyploid taxa, therefore highlighting the potential importance of this evolutionary mechanism in island systems (Obbard *et al*. 2006; Garcia-Verdugo *et al*. 2015). Indeed, while showing signs of population reduction, fragmentation, and local extinction, we found an example of a rare species that still maintains high levels of genetic diversity. External factors such as geographical distance and geographical barriers improved our ability to model genetic distance. Therefore, the classical models should be tested with newer and more integrated versions, including not only geographical distances but also relevant geographical barriers. While addressing oceanic island floras, discrepancies between the hierarchical and stepping stone classical models could be unravelled (Meirmans 2012), contributing to the eventual development of a more unified modelling approach for genetic distance, including also other dimensions such as temporal aspects (Anderson *et al*. 2010).

Finally, this study revealed that *L. watsoniana* populations show considerable genetic diversity, also representing another case in the Azores of pronounced genetic differentiation among populations in different islands. This information on the remaining *Lactuca* populations in the archipelago should be taken into consideration in the design of a recovery plan.

## Sources of Funding

This study was funded by Fundo Regional da Ciência (M3.1.2/F/032/2011) and FEDER funds through the Operational Programme for Competitiveness Factors—COMPETE and by National Funds through FCT—Foundation for Science and Technology under the UID/BIA/50027/2013 and POCI-01-0145-FEDER-006821.

## Contributions by the Authors

E.F.D., participated in sampling, performed genetic analyses, and wrote the first draft; E.F.D and L.S. conceived the idea for the paper; L. S. was involved in the statistical analysis; M.M. was involved in the genetic analysis; H.S. participated in field work. All authors helped revise the manuscript.

## Conflict of Interest Statement

None declared.
